# Cyanidin-3-Glucoside inhibits ethanol-induced invasion of breast cancer cells overexpressing ErbB2

**DOI:** 10.1186/1476-4598-9-285

**Published:** 2010-10-29

**Authors:** Mei Xu, Kimberly A Bower, Siying Wang, Jacqueline A Frank, Gang Chen, Min Ding, Shiow Wang, Xianglin Shi, Zunji Ke, Jia Luo

**Affiliations:** 1 Department of Internal Medicine, University of Kentucky College of Medicine, Lexington, KY 40536, USA; 2 Pathophysiological Department, School of Basic Medicine, Anhui Medical University, Hefei, Anhui, PR China 230032; 3 National Institute for Occupational Safety and Health, Morgantown, West Virginia 26505, USA; 4 Beltsville Agricultural Research Center, U. S. Department of Agriculture, Beltsville, Maryland 20705, USA; 5 Graduate Center for Toxicology, University of Kentucky, 232 Health Sciences Research Building, Lexington, Kentucky 40536, USA; 6 Institute for Nutritional Sciences, Shanghai Institutes for Biological Sciences, Chinese Academy of Sciences, Shanghai, PR China 200031

## Abstract

**Background:**

Ethanol is a tumor promoter. Both epidemiological and experimental studies suggest that ethanol may enhance the metastasis of breast cancer cells. We have previously demonstrated that ethanol increased the migration/invasion of breast cancer cells expressing high levels of ErbB2. Amplification of ErbB2 is found in 20-30% of breast cancer patients and is associated with poor prognosis. We sought to identify agents that can prevent or ameliorate ethanol-induced invasion of breast cancer cells. Cyanidin-3-glucoside (C3G), an anthocyanin present in many vegetables and fruits, is a potent natural antioxidant. Ethanol exposure causes the accumulation of intracellular reactive oxygen species (ROS). This study evaluated the effect of C3G on ethanol-induced breast cancer cell migration/invasion.

**Results:**

C3G attenuated ethanol-induced migration/invasion of breast cancer cells expressing high levels of ErbB2 (BT474, MDA-MB231 and MCF7^ErbB2^) in a concentration dependent manner. C3G decreased ethanol-mediated cell adhesion to the extracellular matrix (ECM) as well as the amount of focal adhesions and the formation of lamellipodial protrusion. It inhibited ethanol-stimulated phosphorylation of ErbB2, cSrc, FAK and p130^Cas^, as well as interactions among these proteins. C3G abolished ethanol-mediated p130^Cas^/JNK interaction.

**Conclusions:**

C3G blocks ethanol-induced activation of the ErbB2/cSrc/FAK pathway which is necessary for cell migration/invasion. C3G may be beneficial in preventing/reducing ethanol-induced breast cancer metastasis.

## Background

Excessive ethanol consumption is associated with an increased risk for breast cancer [1-5]. Epidemiological studies indicate that alcohol consumption is associated with advanced and invasive breast tumors [6,7]. We have previously demonstrated that breast cancer cells or mammary epithelial cells expressing high levels of ErbB2 are sensitive to ethanol-mediated migration/invasion; ethanol stimulates migration/invasion of breast cancers with high ErbB2 levels more robustly than cells expressing lower levels of ErbB2 [8-10]. ErbB2 belongs to the ErbB family of receptor kinases which consists of EGFR, ErbB2, ErbB3 and ErbB4. Among the ErbB family, ErbB2 is most directly related to breast cancer and is implicated in breast cancer metastasis. Amplification of ErbB2 is found in 20-30% of breast cancer patients and is associated with poor prognosis and relapse [11,12].

We sought to identify agents that may ameliorate ethanol's promoting effect on breast cancer cell migration/invasion. Cyanidin-3-glucoside (C3G) is a member of the anthocyanin family which is present in various vegetables and fruits, especially edible berries. C3G is a potent antioxidant and displays anti-cancer properties *in vitro *and *in vivo *[13-18]. Since ethanol exposure causes the accumulation of intracellular oxygen species (ROS) and many biological effects of ethanol are believed to be mediated by ROS, we hypothesize that C3G may inhibit ethanol-induced migration/invasion of breast cancer cells. We examined the effect of C3G on ethanol-mediated migration/invasion of breast cancer cells expressing high levels of ErbB2. We demonstrate here that C3G effectively blocks ethanol-induced cell migration/invasion. We further investigate the effect of C3G on the cell/extracellular matrix (ECM) interaction and the associated ErbB2/cSrc/FAK pathway.

## Materials and methods

### Materials

Human plasma fibronectin was obtained from Chemicon International (Temecula, CA). Anti-paxillin antibody was purchased from Invitrogen Corporation (Carlsbad, CA). Anti-phospho-ErbB2 (Tyr1248) (polyclonal), phospho-p130^Cas ^and ErbB2 (polyclonal) antibodies were purchased from Cell Signaling Technology Inc. (Beverly, MA). Anti-Neu/Her2/ErbB2 (monoclonal), FAK, cSrc, JNK and phospho-Src (Tyr216) antibodies and Protein A/G beads were purchased from Santa Cruz Biotechnology (San Diego, CA). Anti-phospho-Her2/ErbB2 (Tyr1248) (monoclonal) and phospho-FAK (Tyr861) antibodies were purchased from Biosource (Camarillo, CA). Anti-p130^Cas ^antibody was obtained from BD Transduction Laboratory (San Jose, CA). Anti-active JNK antibody was obtained from Promega Corporation (Madison, WI). Phalloidin 488, Alex Fluor-labeled secondary antibodies, Prolong Gold anti-fade reagent and reactive oxygen species detection reagents were obtained from Invitrogen Molecular Probes (Eugene, OR). MTT assay kit was purchased from Roche Molecular Biochemicals (Indianapolis, IN). Matrigel Invasion Chambers were purchased from BD Biosciences (Bedford, MA). Transwell was obtained from Costar Corp. (Acton, MA). C3G was purified from blackberry fruit tissue as previously described [14]. The purity of C3G is greater than 95%. Alcohol (200 Proof) was obtained from Fisher Scientific (Pittsburgh, PA). All other chemicals were obtained from Sigma-Aldrich (St. Louis, MO).

### Cell culture and ethanol exposure

MCF7^ErbB2 ^(MCF7 cells overexpressing ErbB2) and MDA-MB231 breast cancer cells were grown in DMEM medium containing 10% fetal bovine serum (FBS), penicillin (100 U/ml)/streptomycin (100 U/ml), 1 μg/ml hydrocortisone and 10 μg/ml insulin at 37°C with 5% CO_2_. BT474 cells were grown in RPMI 1640 medium containing 10% FBS, penicillin (100 U/ml)/streptomycin (100 U/ml) and 10 μg/ml insulin. A method utilizing sealed containers was used to maintain ethanol concentrations in the culture medium. The containers were placed in a humidified environment and maintained at 37°C with 5% CO_2._

### Cell invasion and migration

Cell invasion was assayed using Matrigel Invasion Chambers (BD Biosciences). Briefly, cells were placed on the upper compartment of invasion chambers and treated with ethanol in the presence or absence of C3G. Culture medium containing 10% FBS was added into the lower compartment of invasion chambers and served as chemoattractants for the cells. Cells were maintained in the invasion chambers for 48 hours. The invaded cells were fixed in 3.7% paraformaldehyde and stained with 0.5% crystal violet in 2% ethanol. Membranes were washed and the dye was eluted with 10% acetic acid. Absorbance was measured at 595 nm using a microtiter platereader (Beckman coulter).

Cell migration was analyzed using a Transwell Migration System (Costar). Briefly, cells were plated into upper chambers (Transwells with 8.0 μm pore size) in serum free medium. The lower compartment of the chamber contained regular medium containing 10% FBS. The chambers were cultured at 37°C in 5% CO_2 _for 12 hours. Migrated cells were fixed and stained with 0.5% crystal violet, followed by dye elution and absorbance measurement as described above.

### Wound healing migration assay

The wound healing migration assay was performed as described previously [14]. MDA-MB231 cells were grown on 35 mm dishes to 100% confluence and then scratched to form a wound using sterile pipette tips. The cells were then treated with ethanol (0 or 400 mg/dl) in the presence or absence of C3G (10 μM) for 24 hours. The images were recorded using a Zeiss Axiovert 40C photomicroscope.

### Analysis of cell adhesion

Cell adhesion to fibronectin was analyzed as described previously [19-21]. Briefly, 96-well cell culture plates were precoated with fibronectin (10 μg/ml) for 60 min at 37°C. Plates were then incubated with 3% BSA in PBS for 30 min to block non-specific binding sites, followed by several washes with PBS. Cells were exposed to ethanol with/without C3G for 48 hours. After exposure, cells (5 × 10^4^/well) were seeded on fibronectin precoated plates, allowing attachment for 1 hour at 37°C with 5% CO_2_. Non-adherent cells were removed by washing with PBS. The attached cells were fixed with 3.7% paraformaldehyde for 10 min, washed 3 times in PBS, and stained with 0.1% crystal violet in 2% ethanol for 10 min. Cells were rinsed with water and dried. Crystal violet was eluted in 10% acetic acid and the absorbance (attached cells) was measured at 595 nm using a microtiter platereader.

### MTT assay

The MTT assay was employed to determine the number of viable cells in culture. Briefly, the cells were plated into 96-well plates and exposed to ethanol with/without C3G for indicated times. After the treatment, 10 μl of MTT reagent was added into each well and the plates were incubated at 37°C for 4 hours. The cultures were solubilized and spectrophotometric absorbance was measured at 595 nm using a microtiter platereader.

### Immunofluorescence microscopy

The procedure for immunofluorescence microscopy has been previously described [22]. Briefly, after treatments, cells were seeded on fibronectin (10 μg/ml) precoated coverslips. Cells were fixed with 3.7% paraformaldehyde for 10 min, washed 3 times in PBS and permeabilized with 0.5% Triton X-100 for 5 min. Cells were blocked with 5% BSA and incubated with primary antibodies for 1 hour. The concentrations of primary antibodies were: phospho-FAK (Tyr861), 1:50; paxillin, 1:800; and phalloidin, 1:200. Following incubation with primary antibodies, cells were washed and treated with Alexa Fluor-labeled secondary antibodies and rinsed several times with PBS. Coverslips were mounted with Prolong Gold anti-fade reagent and immunofluorescence images were examined with a LEICA SP1 inverted confocal microscope. The fluorescent signals were measured with the same pinhole, detector gain and amplifier offset. The focal adhesions were detected by immunostaining for phosphorylated FAK and quantified randomly on 10 or more cells for each treatment condition.

### Immunoprecipitation and immunoblotting

After the treatment of ethanol and/or C3G, cells were trypsinized and seeded on fibronectin (10 μg/ml) precoated dishes allowing attachment for indicated times. Cells were then rinsed twice in cold PBS to remove non-adherent cells. Attached cells were lysed in modified RIPA buffer (150 mM NaCl, 50 mM Tris, 1% NP-40, 0.25% sodium deoxycholate, 1 mM sodium vanadate, 1 mM phenylmethanesulfonyl fluoride (PMSF), 5 μg/ml of aprotinin, and 2 μg/ml of leupeptin). The procedure for immunoprecipitation and immunoblotting has been previously described [10,19]. Briefly, equal amounts of proteins (about 500-800 μg) were incubated with anti-ErbB2, FAK, p130^Cas ^or cSrc antibodies overnight at 4°C, followed by treatment with TrueBlot anti-mouse Ig or anti-rabbit Ig beads (eBioscience, San Diego, CA) for 2 hours at 4°C. Immunoprecipitates were collected by centrifugation at 10,000 g for 5 min at 4°C. Samples were washed five times with RIPA buffer, one time with cold PBS and boiled in sample buffer (187.5 mM Tri-HCl, pH 6.8, 6% SDS, 30% glycerol, 150 mM DTT and 0.03% bromophenol blue). Proteins were resolved in SDS-PAGE and the separated proteins were transferred to nitrocellulose membranes. The membranes were probed with indicated primary antibodies, followed by the appropriate TrueBlot horseradish peroxidase-conjugated secondary antibodies and developed by enhanced chemiluminescence.

### Detection of intracellular reactive oxygen species

Intracellular reactive oxygen species (ROS) levels were measured using the fluorescent dye CM-H_2_DCFDA (Invitrogen Corporation, Carlsbad, CA) as previously described [23]. CM-H_2_DCFDA is converted to a non-fluorescent derivative inside the cells and when oxidized forms a highly fluorescent product by intracellular ROS. Briefly, cells were treated with ethanol with/without C3G or other antioxidants for 48 hours. After the treatment, cells were washed with cold PBS and incubated with 5 μM CM-H_2_DCFDA for 30 min, followed by several additional washes with cold PBS. Cells were trypsinized and transferred into polystyrene round-bottom tubes; intracellular ROS levels were measured with a flow cytometer (FACScalibur, BD Biosciences, San Jose, CA) at an emission wavelength of 525 nm.

### Statistics

Differences among treatment groups were analyzed using analysis of variance (ANOVA). Differences in which *p *was less than 0.05 were considered statistically significant. In cases where significant differences were detected, specific *post-hoc *comparisons between treatment groups were examined with Student-Newman-Keuls tests.

## Results

### C3G inhibits ethanol-enhanced migration/invasion and attachment of breast cancer cells

**Figure 1:**
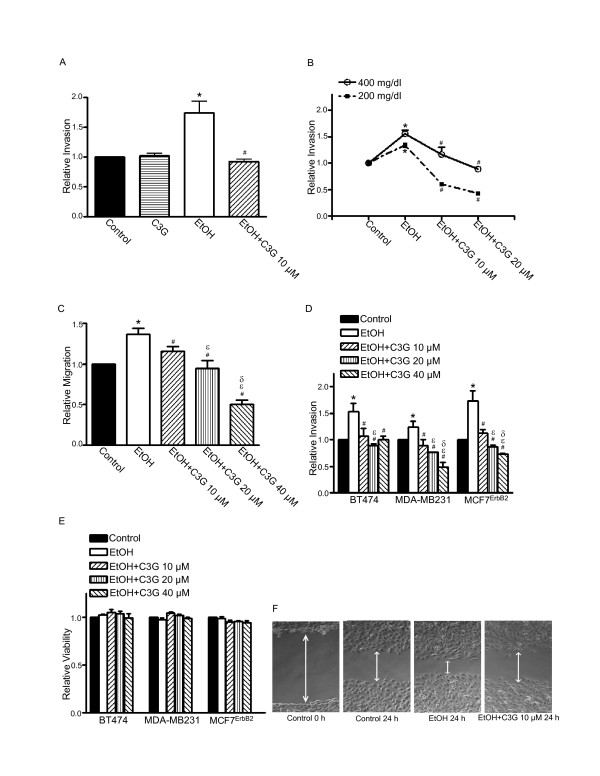
**Effects of C3G on ethanol-mediated invasion/migration of breast cancer cells**. **A**: MCF7^ErbB2 ^cells were plated into the upper compartments of the matrigel invasion chambers and exposed to ethanol (0 or 400 mg/dl) with/without C3G (10 μM) for 48 h. Following the treatment, the invasive potential was assayed as described under the Materials and Methods and presented relative to untreated controls. **B**: MCF7^ErbB2 ^cells were exposed to ethanol (0, 200 or 400 mg/dl) with/without C3G (10 or 20 μM) for 48 h. The invasive potential was assayed as described above. **C**: MCF7^ErbB2 ^cells were plated into the upper compartments of the migration chamber and exposed to ethanol (0 or 400 mg/dl) with/without C3G (10, 20 or 40 μM) for 12 h. The migration was analyzed as described under the Materials and Methods and presented relative to untreated controls. **D**: BT474, MDA-MB231 or MCF7^ErbB2 ^cells were exposed to ethanol (0 or 400 mg/dl) with/without C3G (10, 20 or 40 μM) for 48 h. Their invasion potential was evaluated as described above. **E**: BT474, MDA-MB231 or MCF7^ErbB2 ^cells were exposed to ethanol (0 or 400 mg/dl) with/without C3G (10, 20 or 40 μM) for 48 h and cell viability was determined with MTT assay. The number of viable cells was presented relative to untreated controls. Each datum point was the mean ± SEM of three independent experiments. * denotes a statistically significant difference from untreated controls. # denotes a significant difference from ethanol-treated groups. ε denotes a significant difference from ethanol- and C3G (10 μM)-treated groups. δ denotes a significant difference from ethanol- and C3G (20 μM)-treated groups. **F**: MDA-MB231 cells were exposed to ethanol (0 or 400 mg/dl) with/without C3G (10 μM) for 24 h and cell migration was determined by wound healing migration assay as described under the Materials and Methods.

We have previously demonstrated that the effect of ethanol on the migration/invasion of breast cancer cells is positively associated with their expression levels of ErbB2 [8-10]. The current study confirmed the finding and showed that ethanol increased the migration/invasion of MCF7^ErbB2^, BT474 and MDA-MB231 breast cancer cells (Figure 1). C3G (10-40 μM) significantly inhibited ethanol-enhanced migration/invasion of MCF7^ErbB2 ^and MDA-MB231 in a concentration dependent manner (Figures 1B-D). C3G-mediated inhibition was statistically different among the three C3G concentrations tested. The effect of C3G on BT474 cells, however, was not dose-dependent (Figure 1D). C3G alone at 10 μM did not affect the invasion of MCF7^ErbB2 ^cells (Figure 1A). For BT474 and MDA-MB231 cells, C3G alone produced a modest but statistically significant inhibition of cell invasion (data not shown). The inhibitory effect of C3G on ethanol-induced cell migration was confirmed by a wound healing migration assay (Figure 1F). The MTT assay showed that even 40 μM C3G did not affect the viability of BT474, MDA-MB231 and MCF7^ErbB2 ^cells (Figure 1E). However, at 100 μM or above, C3G did decrease cell viability (data not shown).

**Figure 2:**
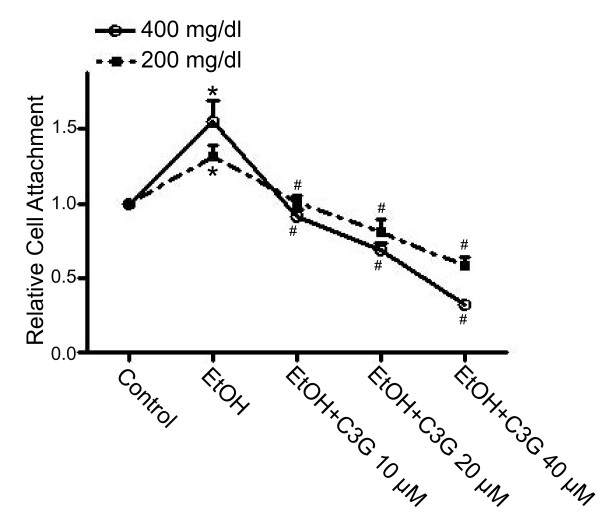
**Effects of C3G on ethanol-mediated adhesion of breast cancer cells**. MCF7^ErbB2 ^cells were treated with ethanol (0, 200 or 400 mg/dl) with/without C3G (10, 20 or 40 μM) for 48 h, and then equal amounts of cells were seeded on fibronectin-coated culture wells, allowing attachment for 1 h. The number of adherent cells was determined as described under the Materials and Methods and presented relative to untreated controls. Each datum point was the mean ± SEM of three independent experiments. * denotes a statistically significant difference from untreated controls. # denotes a significant difference from ethanol-treated groups.

The adhesion of cancer cells to ECM or cell/ECM interaction is an important step of metastasis. We have previously demonstrated that ethanol enhances the adhesion of breast cancer cells to fibronectin, an essential protein in the ECM [19]. Ethanol did not affect the attachment of breast cancer cells to poly-lysine (data not shown). We examined the effect of C3G on ethanol-mediated cell adhesion to fibronectin. MCF7^ErbB2 ^cells were pretreated with ethanol with/without C3G for 48 hours, then the cells were seeded on fibronectin precoated plates and allowed to attach for 1 hour in the presence/absence of ethanol and/or C3G. As shown in Figure 2, ethanol increased the adhesion of MCF7^ErbB2 ^cells to fibronectin and C3G significantly inhibited ethanol-enhanced adhesion in a concentration-dependent manner. C3G alone (10-20 μM) did not affect cell adhesion (data not shown). C3G similarly inhibited ethanol-induced adhesion of MDA-MB231 cells to the ECM (data not shown).

### C3G attenuates ethanol-stimulated ErbB2 signaling

**Figure 3:**
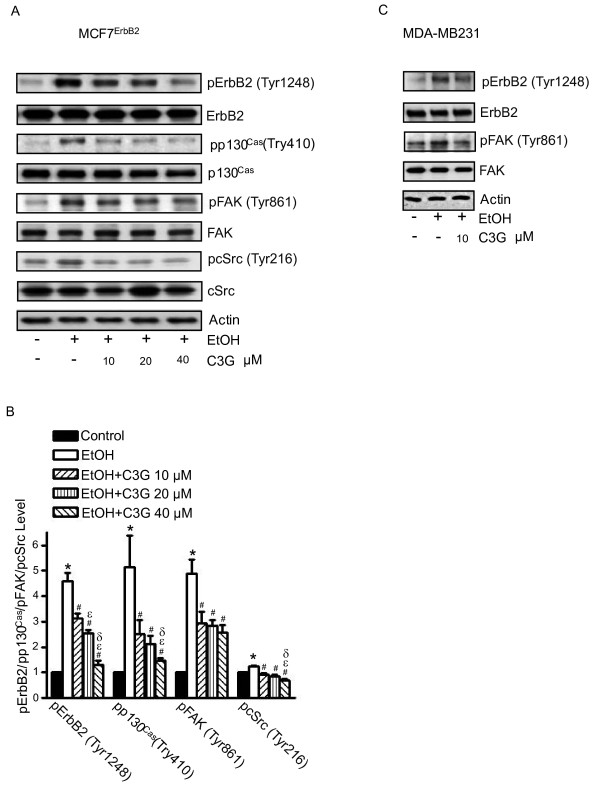
**Effects of C3G on ethanol-mediated phosphorylation of ErbB2, cSrc, FAK and p130^Cas^**. **A**: MCF7^ErbB2 ^cells were treated with ethanol (0 or 400 mg/dl) with/without C3G (10, 20 or 40 μM) for 48 h. Cells were seeded on fibronectin-coated culture wells for 3 h and then harvested for analysis of the phosphorylation of ErbB2, FAK, p130^Cas ^and cSrc with immunoblotting. The expression of actin served as a loading control. **B**: The relative expression of phosphorylated ErbB2, FAK, p130^Cas ^and cSrc was determined by densitometry and normalized to the expression of actin. * denotes a statistically significant difference from untreated controls. # denotes a significant difference from ethanol-treated groups. ε denotes a significant difference from ethanol- and C3G (10 μM)-treated groups. δ denotes a significant difference from ethanol- and C3G (20 μM)-treated groups. **C**: The phosphorylation of ErbB2 and FAK in MDA-MB231 cells was analyzed as described above. The experiment was replicated three times.

**Figure 4:**
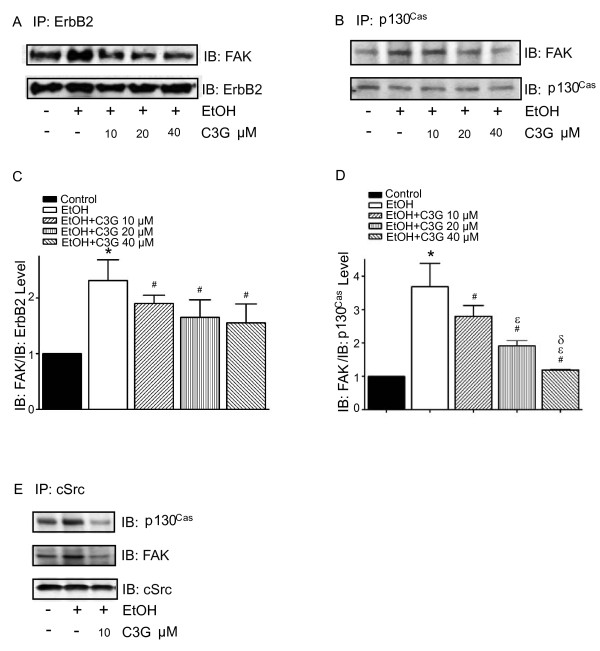
**Effects of C3G on the interaction among ErbB2, FAK, cSrc and p130^Cas^**. MCF7^ErbB2 ^cells were treated with ethanol (0 or 400 mg/dl) with/without C3G (10, 20 or 40 μM) for 48 h. Cells were plated on fibronectin-coated culture wells. **A**: After 1 h of attachment on fibronectin, cell lysates were collected and immunoprecipitated (IP) with an anti-ErbB2 antibody, then immunoblotted (IB) with either an anti-FAK or anti-ErbB2 antibody. **B**: After 3 h of attachment, cell lysates were IP with an anti-cSrc antibody and IB with either an anti-p130^Cas^, FAK or cSrc antibody. **C and D**: The association between ErbB2 and FAK (panel A) and the association between FAK and p130^Cas ^(panel B) was quantified by densitometry. * denotes a statistically significant difference from untreated controls. # denotes a significant difference from ethanol-treated groups. ε denotes a significant difference from ethanol- and C3G (10 μM)-treated groups. δ denotes a significant difference from ethanol- and C3G (20 μM)-treated groups. **E**: After 3 h of attachment, cell lysates were IP with an anti-p130^Cas ^antibody and IB with either an anti-FAK or anti-p130^Cas ^antibody. The experiment was replicated three times.

**Figure 5:**
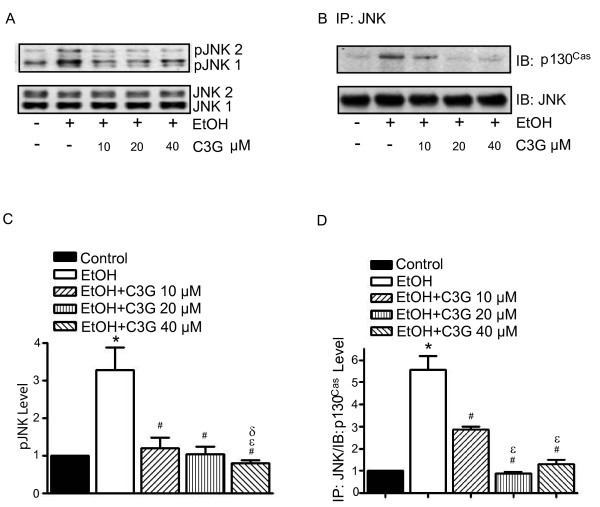
**Effects of C3G on ethanol-induced activation of JNKs**. MCF7^ErbB2 ^cells were treated with ethanol (0 or 400 mg/dl) with/without C3G (10, 20 or 40 μM) for 48 h. Cells were seeded on fibronectin-coated culture wells for 3 h. **A**: Cell lysates were collected and analyzed for the phosphorylation/expression of JNKs with immunoblotting. **B**: Cell lysates were IP with an anti-JNK antibody and IB with either an anti-p130^Cas ^or anti-JNK antibody. The experiment was replicated three times. **C and D**: The phoshorylation of JNKs and the association between JNKs and p130^Cas ^were quantified by densitometry. * denotes a statistically significant difference from untreated controls. # denotes a significant difference from ethanol-treated groups. ε denotes a significant difference from ethanol- and C3G (10 μM)-treated groups. δ denotes a significant difference from ethanol- and C3G (20 μM)-treated groups.

We have previously shown that ethanol increased the phosphorylation of ErbB2 at Tyr1248 [19]. In this study, we examined the effect of C3G on ethanol-mediated ErbB2 phosphorylation. MDA-MB231 and MCF7^ErbB2 ^cells were pretreated with ethanol with/without C3G for 48 hours, then cells were seeded into fibronectin precoated dishes, allowing attachment for 3 hours. As shown in Figure 3, ethanol drastically increased the phosphorylation of ErbB2 [p-ErbB2(Tyr1248)] in these cells. The addition of C3G attenuated ethanol-stimulated p-ErbB2(Tyr1248) in a concentration-dependent manner. The cSrc/FAK pathway plays an important role in ErbB2-regulated migration/invasion of breast cancer cells [24]. FAK is a substrate of cSrc and FAK Tyr861 is a major site of phosphorylation by cSrc. As shown in Figure 3, ethanol increased the levels of p-FAK(Tyr861) and p-cSrc(Tyr216). C3G attenuated ethanol-induced p-FAK(Tyr861) and p-cSrc(Tyr216). The activation and phosphorylation of cSrc/FAK is critical for triggering its downstream signaling and for recruiting proteins to the focal adhesion sites. p130^Cas^, an adaptor protein, binds to the C-terminal site of FAK, forming a dock site for Crk; p130^Cas^/Crk interaction induces the activation of small GTPases and JNKs, promoting membrane protrusion and cell migration [25,26]. The phosphorylation of p130^Cas ^is regulated by FAK and cSrc [27]. We demonstrated that ethanol induced the phosphorylation of p130^Cas ^[p-p130^Cas^(Tyr410)], and C3G blocked ethanol-induced p-p130^Cas^(Tyr410) (Figure 3A). We further examined the effect of C3G on the interaction among ErbB2, cSrc, FAK and p130^Cas^. MCF7^ErbB2 ^cells were treated with ethanol or with/without C3G for 48 hours and seeded on fibronectin for 1 or 3 hours. As shown in Figure 4, ethanol increased the association between ErbB2/FAK, FAK/cSrc, FAK/p130^Cas ^and cSrc/p130^Cas^. C3G abolished the interaction among these proteins (Figure 4). These data indicated that C3G inhibited the ethanol-activated ErbB2/cSrc/FAK pathway.

c-Jun N-terminal kinases (JNKs), a member of mitogen-activated protein kinases (MAPKs), regulate cell migration/invasion [28]. We have previously demonstrated that JNKs are essential for ethanol-mediated cell invasion/migration [10]. JNK activation is regulated by p130^Cas ^[27]. C3G inhibited ethanol-induced JNK phosphorylation and p130^Cas^/JNK association in MCF7^ErbB2 ^cells (Figure 5).

### C3G inhibits ethanol-induced formation of lamellipodia and focal adhesions

**Figure 6:**
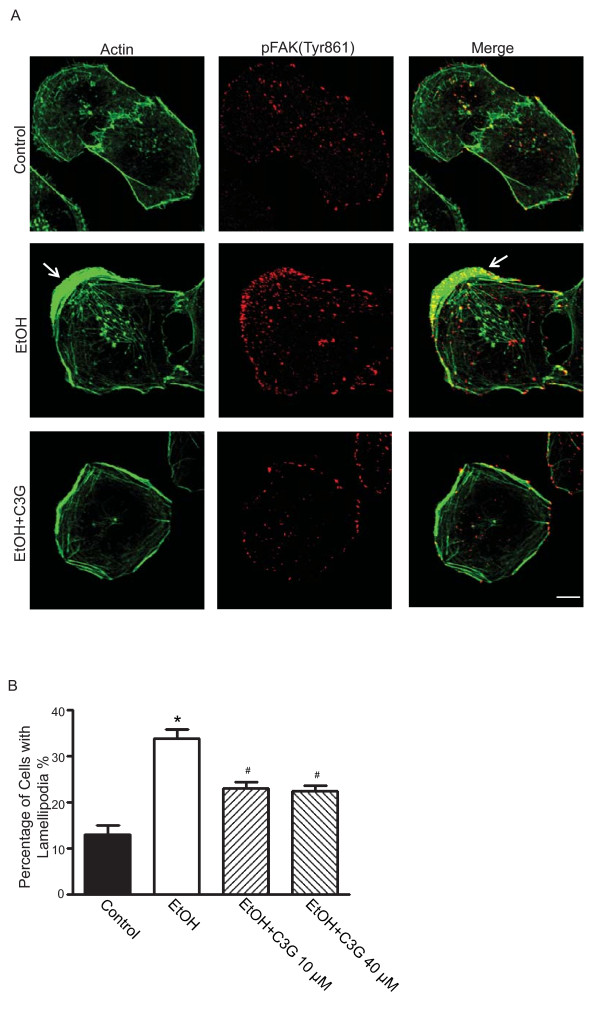
**Effects of C3G on the development of lamellipodia**. MDA-MB231 cells were treated with ethanol (0 or 400 mg/dl) with/without C3G (10 or 40 μM) for 48 h. Cells were seeded on fibronectin-coated coverslips for 3 h. **A**: The expression of actin (Alexa Fluor 488 Phalloidin) and phosphorylated FAK (Tyr 861) (Alexa Flour 594) were detected with immunofluorescent staining. The arrow indicates lamellipodia. Scale bar = 5 μm. **B**: Cells with extended leading areas (lamellipodia) were counted in ten randomly selected fields in each treatment group. The percentage of cells with lamellipodia was determined. The experiment was replicated three times. * denotes a significant difference from untreated controls. # denotes a significant difference from ethanol-treated groups.

**Figure 7:**
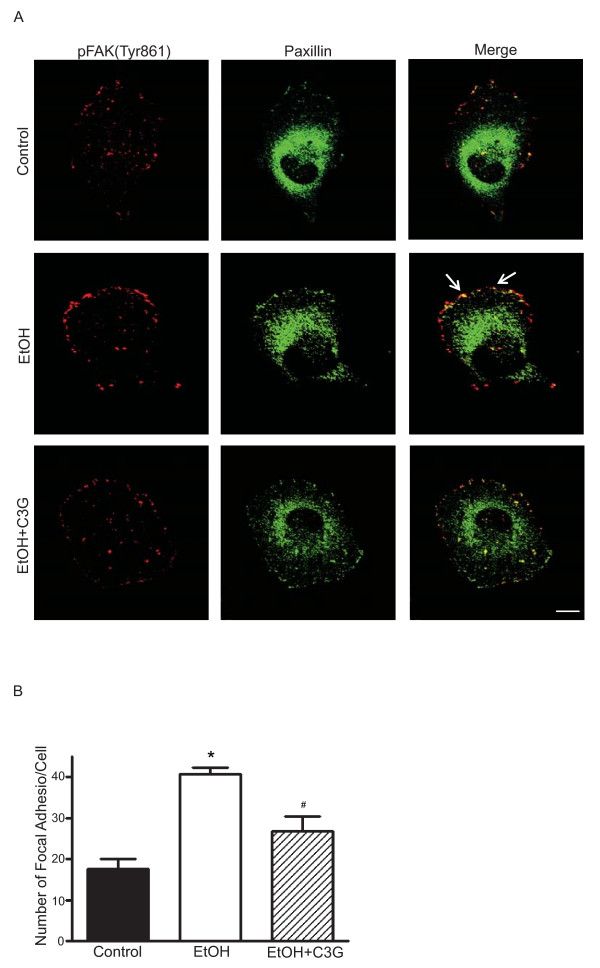
**Effects of C3G on ethanol-mediated formation of focal adhesions**. MDA-MB231 cells were treated with ethanol (0 or 400 mg/dl) with/without C3G (40 μM) for 48 h. Cells were seeded on fibronectin-coated coverslips for 3 h. **A**: The expression of paxillin (Alexa Fluor 488) and phosphorylated FAK (Tyr861) (Alexa Fluor 594) were detected by immunofluorescent staining. Arrows indicate the co-localization of p-FAK (Tyr861) and paxillin. Scale bar = 5 μm. **B**: Focal adhesions were counted randomly on 10 or more cells. The number of focal adhesions per cell was calculated. Each datum point was the mean ± SEM of three independent experiments. * denotes a significant difference from untreated controls. # denotes a significant difference from ethanol-treated groups.

The initiation of cell migration requires the development of membrane protrusion, the lamellipodium and the assembly of dynamic focal adhesions with the ECM [29]. We sought to determine whether C3G affected the formation of the lamellipodium and focal adhesions. We used MDA-MB231 cells for this experiment because these cells displayed more prominent lamellipodium and focal adhesions during the migration process. Figure 6A shows that actin filament distribution was concentrated at the leading edge/lamellipodia in ethanol-treated MDA-MB231 cells. Ethanol caused an approximate 3-fold increase in the number of lamellipodia (Figure 6B). C3G inhibited ethanol-induced lamellipodia formation; however, the inhibition was not concentration-dependent and C3G at 10 or 40 μM had a similar effect (Figure 6B). We demonstrated an accumulation of p-FAK(Tyr861) at the leading area in ethanol-treated cells (Figures 6A and 7A). Ethanol also caused redistribution of paxillin, and more paxillin was localized at the leading edge following ethanol exposure (Figure 7A). Since the activation of FAK leads to the recruitment of paxillin and p130^Cas ^to focal adhesion sites [27,30], we examined the effect of ethanol on focal adhesions. Ethanol enhanced the assembly of focal adhesions and C3G significantly inhibited ethanol-induced formation of focal adhesions (Figure 7B).

### C3G scavenges ethanol-induced accumulation of reactive oxygen species (ROS)

**Figure 8:**
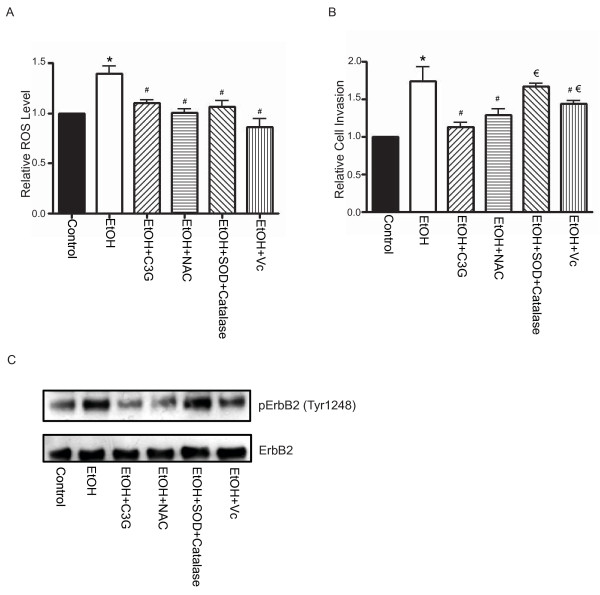
**Effects of C3G and antioxidants on ethanol-induced ROS generation, cell invasion and ErbB2 phosphorylation**. **A**: MCF7^ErbB2 ^cells were exposed to ethanol (0 or 400 mg/dl) with/without C3G (10 μM), SOD (50 U/ml)/catalase (200 U/ml), NAC (5 mM) or vitamin C (20 μM) for 48 h. Intracellular ROS levels were measured by flow cytometry as described under the Materials and Methods. **B**: The invasive potential of MCF7^ErbB2 ^cells was evaluated as described above and expressed relative to untreated controls. **C**: The phosphorylation of ErbB2 in MCF7^ErbB2 ^cells was analyzed with immunoblotting. The experiment was replicated three times. * denotes a statistically significant difference from untreated controls. # denotes a significant difference from ethanol-treated groups. # denotes a significant difference from ethanol- and C3G-treated groups.

Ethanol causes intracellular accumulation of reactive oxygen species (ROS) and induces oxidative stress [10,31]. Since C3G is a potent antioxidant, the inhibitory effect of C3G on ethanol-induced migration/invasion may be mediated by its antioxidant property. We evaluated the effect of other antioxidants at concentrations that had a similar ROS scavenging capacity as C3G. Superoxide dismutase (SOD) is a scavenger for O_2_^• ^and catalase is a scavenger for hydrogen peroxide (H_2_O_2_). N-aceytlcysteine (NAC) (5 mM), vitamin C (20 μM) and SOD (50 U/ml) plus catalase (200 U/ml) had approximately the same antioxidant effect as C3G (10 μM) (Figure 8A). As shown in Figure 8B, C3G most effectively inhibited ethanol-enhanced invasion of breast cancer cells; NAC and vitamin C also provided significant inhibition, but to a lesser extent. On the other hand, SOD plus catalase had little effect on ethanol-enhanced cell invasion. A similar result regarding the effect of C3G and other antioxidants on ethanol-induced ErbB2 phosphorylation was observed (Figure 8C).

## Discussion

### C3G as a potent agent to alleviate ethanol-induced cell migration/invasion

In search for better chemopreventive or chemotherapeutic agents, we isolated a natural antioxidant cyanidin-3-glucoside (C3G) from blackberries [14,32]. C3G is a member of the anthocyanin family which is present in various vegetables and fruits, especially edible berries. We have confirmed C3G's antioxidant property [14,23]. C3G has been implicated in some beneficial health actions including reducing age-associated oxidative stress, improving cognitive brain function, as well as anti-diabetic, anti-inflammation, anti-atherogenic and anti-obesity activity [33]. C3G exhibits anti-cancer properties in various *in vitro *and animal models of carcinogenesis and tumor development; the effects of C3G include the inhibition of tumor cell proliferation and the attenuation of cell migration/invasion as well as metastasis *in vivo *[13-18].

We demonstrate here that C3G inhibits ethanol-mediated migration/invasion in cells expressing high levels of ErbB2. C3G has a greater inhibitory effect on the invasion of cells treated with 200 mg/dl ethanol compared to 400 mg/dl (Figure 1B); the underlying mechanism is unclear. C3G is effective at 10 μM, a concentration that is lower than previously reported for its anti-cancer effects. C3G inhibits the migration/invasion of A549 lung cancer cells at 40-100 μM [14,34]. It is unlikely that C3G-mediated inhibition of tumor cell migration/invasion in this study results from decreased cell viability. C3G up to 40 μM does not affect the viability of breast cancer cells (Figure 1D). It is reported that C3G at 100 μM fails to reduce the viability of A549 lung cancer cells [34]. However, at 20 μM, C3G significantly decreases the viability of HS578T human breast cancer cells [13]. Thus, cells apparently display differential sensitivity to C3G. For BT474 cells, C3G does not have a dose-dependent inhibitory effect. It is likely BT474 cells are more sensitive to C3G and the concentration may need to be lower in order to see the dose-dependent inhibition.

Consistent with its effects on migration/invasion, C3G affects early events associated with cell motility. C3G inhibits ethanol-mediated cell adhesion to the ECM, formation of focal adhesions and development of lamellipodia. These events are prerequisites for cell migration/invasion. These results suggest that ethanol-induced migration/invasion is initiated by tumor cell/ECM interaction and C3G blocks this interaction.

### C3G and ethanol-stimulated cell signaling

Ethanol-stimulated tumor cell/ECM interaction may be initiated by its effect on ErbB2 activity. Ethanol increases the phosphorylation of ErbB2 and enhances the adhesion of breast cancer cells with high levels of ErbB2 to the ECM as well as the assembly of focal adhesions in these cells [19]. These effects were not observed in breast cancer cells expressing low levels of ErbB2.

FAK is a critical regulator of cell/ECM interaction and is strongly implicated in tumor aggressiveness [35,36]. It has been shown that FAK is essential for ErbB2/ErbB3-induced oncogenesis and breast cancer invasion [37]. The phosphorylation of FAK at Tyr 861 plays an important role in the invasion of breast cancer cells [38]. FAK is a substrate of cSrc. The activation of ErbB2 recruits cSrc and FAK, resulting in the phosphorylation of cSrc at Tyr 216 and FAK at Tyr 861 in breast cancer cells [39,40]. An ErbB2 inhibitor Tyrphostin (AG825) abolishes the ethanol-stimulated interaction between ErbB2 and FAK, as well as the adhesion of breast cancer cells to the ECM [41]. It appears C3G targets ErbB2 since C3G is able to inhibit ethanol-mediated phosphorylation of ErbB2, cSrc and FAK. Additionally, it inhibits the ethanol-mediated interaction among these proteins.

Previously we have shown that JNK activation is required in ethanol-induced migration/invasion of breast cancer cells [10]. It was reported that JNK activation during cell migration is mediated by p130^Cas^/Crk coupling or JSAP1 (JNK/stress-activated protein kinase-associated protein 1)[42,43]. p130^Cas ^(Crk-associated substrate) is an adaptor protein and it binds to and is phosphorylated by FAK in a FAK/cSrc dependent manner [27]. Activation of p130^Cas ^leads to recruitment of Crk to form an adaptor complex which results in activation of Rac1 and JNKs. We show here that ethanol increases the association of p130^Cas ^with FAK/cSrc and the phosphorylation of p130^Cas^. Ethanol also promotes the association between p130^Cas ^and JNKs. C3G inhibits ethanol-mediated p130^Cas^/JNK interaction (Figure 5). Together, our results indicate that ethanol activates p130^Cas ^and JNKs through the ErbB2/cSrc/FAK pathway. Blocking ErbB2/cSrc/FAK signaling by C3G inhibits ethanol-mediated activation of JNKs which is necessary for cell migration/invasion. At times the effect of C3G on cell signaling components, such as FAK, cSrc and JNK, is not dose-dependent, which is not entirely consistent with its effect on ethanol-induced cell migration/invasion. This suggests that the mechanism of ethanol-induced cell migration/invasion is complex and probably multiple signaling pathways are involved.

### Antioxidant property of C3G

Ethanol exposure causes the accumulation of intracellular ROS [10,23]. The antioxidant property of C3G is confirmed in this study and we show that C3G effectively blocks ethanol-induced ROS production in breast cancer cells. ROS is reported to be involved in the activation of EGFR [44]. To evaluate the involvement of ROS, we compared the effect of C3G with other antioxidants. We have titrated antioxidants and identified concentrations for these antioxidants to produce a ROS scavenging capacity similar to C3G at 10 μM. Although these antioxidants have a similar capacity of scavenging ROS, they are less effective in alleviating ethanol-induced cell invasion and ErbB2 phosphorylation. Broad antioxidants, such as NAC and vitamin C, display modest inhibition on ethanol-induced cell invasion and ErbB2 phosphorylation, but to a lesser extent compared to C3G. More specific antioxidants, SOD (for superoxide) plus catalase (for hydrogen peroxide) fail to inhibit ethanol-stimulated invasion and ErbB2 phosphorylation. These data suggest that although the antioxidant property may be involved, C3G may also act through other mechanisms to regulate ErbB2 signaling and subsequent migration/invasion. We have previously shown that C3G is able to reverse ethanol-induced inhibition of neurite outgrowth in neuronal cells; however, its antioxidant property is minimally involved [23].

C3G has drawn increasing attention because of its potential anti-cancer properties. A recent animal study investigates the pharmacokinetics of C3G and demonstrates that pharmacologically relevant concentrations of C3G are achievable *in vivo *through oral administration or intravenous injection in mice without apparent adverse effects [45]. Further analysis suggests that concentrations required for *in vivo *action of C3G could be much lower than that of *in vitro *[14,45]. In future studies, we will evaluate the effect of C3G on ethanol-induced tumor promotion in animal models. C3G may offer a *novel *avenue for treating alcohol-related disorders.

## Abbreviations

C3G: Cyanidin-3-glucoside; ECM: extracellular matrix; FAK: focal adhesion kinase; IP: Immunoprecipitation; JNKs: c-Jun N-terminal kinases; NAC: N-acetyl-cysteine; ROS: reactive oxygen species; SOD: superoxide dismutase.

## Competing interests

The authors declare that they have no competing interests.

## Authors' contributions

MX carried out the biochemical studies and participated in all experiments in this study. KB, JF and GC participated in assays for cell treatment, immunoblotting and ethanol exposure paradigm. MD, SW, XS, ZK and JL conceived of the study, and participated in its design and coordination and helped to draft the manuscript. All authors read and approved the final manuscript.
